# The possible role of intravenous lipid emulsion in the treatment of chemical warfare agent poisoning

**DOI:** 10.1016/j.toxrep.2015.12.007

**Published:** 2016-01-18

**Authors:** Arik Eisenkraft, Avshalom Falk

**Affiliations:** a NBC Protection Division, IMOD, Israel; b Israel Defense Forces Medical Corps, Israel; c The Institute for Research in Military Medicine, The Faculty of Medicine, The Hebrew University, Jerusalem, Israel

**Keywords:** Intravenous lipid emulsion, Organophosphates, Sulfur mustard, Antidotes, Poisoning, Chemical Warfare agents

## Abstract

Organophosphates (OPs) are cholinesterase inhibitors that lead to a characteristic toxidrome of hypersecretion, miosis, dyspnea, respiratory insufficiency, convulsions and, without proper and early antidotal treatment, death. Most of these compounds are highly lipophilic. Sulfur mustard is a toxic lipophilic alkylating agent, exerting its damage through alkylation of cellular macromolecules (e.g., DNA, proteins) and intense activation of pro-inflammatory pathways. Currently approved antidotes against OPs include the peripheral anticholinergic drug atropine and an oxime that reactivates the inhibited cholinesterase. Benzodiazepines are used to stop organophosphate-induced seizures. Despite these approved drugs, efforts have been made to introduce other medical countermeasures in order to attenuate both the short-term and long-term clinical effects following exposure. Currently, there is no antidote against sulfur mustard poisoning. Intravenous lipid emulsions are used as a source of calories in parenteral nutrition. In recent years, efficacy of lipid emulsions has been shown in the treatment of poisoning by fat-soluble compounds in animal models as well as clinically in humans. In this review we discuss the usefulness of intravenous lipid emulsions as an adjunct to the in-hospital treatment of chemical warfare agent poisoning.

## 1 Introduction

Organophosphates (OPs) are cholinesterase inhibitors, that are widely used as pesticides, but still represent a major health problem in the world over [37], [38]. A group of OP compounds developed as chemical nerve agents have been used in a variety of international conflicts and terror events [43], [122]. Despite international efforts to outlaw their use for this purpose, they are still being used against enemy forces and innocent civilians [104]. Recently we have witnessed the devastating consequences of using the nerve agent sarin in the Syrian conflict [43], [116], [133].

OP poisoning leads to a characteristic toxidrome that includes muscarinic, nicotinic and central nervous system signs and symptoms [104], [116], [122]. Without proper antidotal treatment administered immediately after exposure, and depending on the dose, victims may suffer from convulsions, respiratory failure and ultimately, death. Currently accepted antidotal treatment includes the anticholinergic drug atropine, and a reactivator from the oxime family. Benzodiazepines are added to terminate seizures [43], [122]. However, it is evident that even when given early after poisoning, the response to these antidotes is not optimal and does not prevent long-term emotional, neurological and cognitive deficiencies occurring in subjects surviving the acute poisoning. Considerable effort has focused on finding more efficient medical countermeasures [42], [113], [150], [151].

Most of the OPs are lipophilic compounds, and as such, may remain in body tissues, especially fat, for long periods of time, mandating a prolonged medical observation time following initial antidotal treatment [38], [39], [94], [108].

Sulfur mustard is a toxic lipophilic alkylating agent widely used in the past as a chemical warfare agent (CWA) [68], [75], and recently reported to be used by the Islamic State Jihadist group ISIS [128]. It exerts its damage through alkylation of cellular macromolecules (e.g., DNA and intra- and extra-cellular proteins) and intense activation of pro-inflammatory pathways [68]. Following exposure, an on-going worsening process of vesication appears, depending on the extent of exposure and whether the victim was decontaminated in a timely manner [68]. The systems most affected are the lungs and airways, skin, and mucous membranes including the eyes. It takes several hours for many of the signs and symptoms to appear [53], [68]. Once absorbed into the tissues, there are no currently available medical countermeasures to prevent the injury—apart from diminishing the extent of the injury with corticosteroids and non-steroidal anti-inflammatory drugs (NSAIDs), and certain treatment adjuncts aimed at reducing the time-to-healing of local injuries [32], [53].

Intravenous lipid emulsions (ILE) were introduced in the early 1960’s as an energy substrate and calorie source containing essential fatty acids, given parenterally as a nutritional supplement in patients with major injury, infection or nutritional depletion [14], [99]. It is widely used in neonatal intensive care, where infants are frequently dependent on intravenous nutrition in the early weeks of life [137]. For more than a decade, ILE were shown to be effective in the treatment of poisonings by fat-soluble compounds, especially local anesthetics. ILE has been shown to rapidly reverse the clinical toxicity induced by a variety of compounds with diverse kinetics and mechanisms of action [17], [20], [21], [23], [117], [142], [143], [145], [149].

Recently, Zhou et al. [154] proposed to use a combination of ILE and charcoal hemoperfusion in patients with severe OP poisoning. They suggest that with this strategy, care-givers can remove the OP, decrease the amount of antidotes needed, reduce possible side-effects of the drugs, and meanwhile provide an additional energy substrate for the victims. We have found no evidence in the literature on the use of ILE in the treatment of sulfur mustard injury.

In this review we will discuss the potential role of ILE as an adjunct to the in-hospital treatment of CWA poisoning.

## 2 Proposed mechanisms of action

The exact mechanisms by which ILE exert their beneficial effects are not fully understood, and several have suggested synergistic effects of several mechanisms [47], [48], [61]. The mechanisms of action can be divided into intravascular, membrane, and intracellular effects [149]. The original theory explaining the mechanism of lipid rescue was that of “lipid sink”, suggesting sequestration of lipophilic compounds to an expanded intravascular lipid phase, extracting the offending agent from the target tissue, and reversing the toxicity [61], [117], [118], [143], [149]. In support of this theory, toxic drug levels were shown to decrease more rapidly in tissues following the administration of ILE [35], [63], [98], [144], [148], [149], and intravenous lipid emulsions were shown to bind lipid-soluble drugs in vitro [51], [89]. Other hypotheses relate to the mechanism by which ILEs facilitate cardiac rescue from drug poisoning. These include (1) increasing myocardial energy substrate delivery and a direct cardiotonic effect of ILE on the poisoned heart, thus improving cardiac function (a so-called metabolic effect) [7], [57], [117], [126], [130], [143], [149], and (2) an effect of ILE on calcium ion channels through high levels of long-chain fatty acids, leading to increased cardiomyocyte calcium and positive inotropic effect which improves the heart contractility [54], [66], [105]. The latter theory seemed unlikely in view of evidence of the inhibitory effects of fatty acids on Ca^2+^ ion intake by neurons [48]. Recently, the cardioprotective action of the long-chain fatty acids in Intralipd^®^ was found to involve Ca^2+^-homeostasis and rescue signaling pathways that regulate the opening of the mitochondrial permeability transition pore (mPTP) [103], [112]. This activity requires fatty acid metabolism and involves production of reactive oxygen species (ROS) by the mitochondria which, in turn, activates rescue pathways [81], [134]. The relative contribution of the “lipid sink” and other mechanisms to the beneficial effect of ILE on systemic bupivacaine toxicity has been studied using physiologically-based pharmacokinetic and pharmacodynamic (PBPK/PD) modeling [131]. Pharmacokinetic analysis has shown that the amount of bupivacaine sequestered from the heart and brain tissues by standard ILE infusion is too low to account for reversal of toxicity, suggesting that additional mechanisms must be involved [76]. A later study, combining dose-response of the effect of ILE in bupivacaine-poisoned rats with PBPK/PD modeling, has shown that ILE exerts its beneficial effect by three mechanisms operating in concert: lipid scavenging of the drug, a volume effect, and the cardiotonic effect, which was found to be essential for reversal of toxicity [46]. Support for the theory suggesting combined action of these mechanisms is found in the recent detailed mechanistic study by Fettiplace et al. [47]. This study suggested that the “lipid sink” concept is inaccurate, and replaced it with the more accurate “lipid shuttle” mechanism [48]. Additional mechanisms besides lipid sequestration extend the range of action of ILE to less lipophilic compounds.

## 3 The use of ILE in over-dose and poisonings

In recent years, several review articles summarized current experience in animal models and in humans [17], [20], [23], [70], [100], [117], [149], showing repeatable positive effects, suggesting a role for ILE as an antidote for poisoning by lipophilic compounds. Efforts have been initiated to collect all data in a global registry (e.g., http://www.lipidrescue.org and http://www.lipidregistry.org) [23], [117], [149]. In the recent LIPAEMIC report, Cave et. al. [24] summarized the results from three years of operation (2009–2012) of the lipid rescue registry. The first successful use of ILE in a clinical setting of rescue was in 2006 in a patient with bupivacaine-induced cardiovascular collapse [115]. Since then, the list of lipophilic drugs and compounds for which ILE therapy seems to be helpful has expanded [8], [17], [117], [145], [149], [155], to include a number of beta-blockers [19], [21], [33], [58], [127], calcium channel blockers [7], [26], [130], a variety of psychotropic drugs [6], [49], [52], [57], [124], [147], muscle relaxants and macrocytic lactones [72].

Han et al. [56] described a case poisoning in a 52 years old man by glyphosate-surfactant (GlySH), a herbicide consisting of the water-soluble glyphosate and an amphiphilic surfactant [25]. He was admitted with impaired consciousness, hypotension and bradycardia. He was mechanically ventilated, treated with vasopressors and fluids, and had a gastric lavage. However, two and a half hours after initiation of treatment, he remained in a critical condition. At this point the caregivers decided to use 20% ILE. Shortly after his condition stabilized, he was extubated, and several days later was discharged with no sequelae. Mahendrakar et al. [84] reported another case of GlySH ingestion in a 35 years old man. He developed cardiovascular shock and renal failure. He too was treated with fluids, vasopressors, veno-venous hemodiafiltration and 20% ILE. Though this combined treatment was given for a period of several days before clinical improvement was achieved, the patient survived and was discharged home with no reported sequelae. Circulatory shock is a major cause of death in GlySH poisoning, usually refractory to fluid resuscitation and vasopressors [114]. There is no specific antidote to GlySH. It is a water-soluble compound, in which the added surfactant is capable of solubilizing lipids [153]. Thus, ILE may probably reduce the effect of GlySH poisoning by lowering free surfactant concentration, thus preventing its cardiovascular toxicity [56].

Several organizations have included ILE therapy in their guidelines for the treatment of systemic toxicity from local anesthetics, though it is still defined as a Class IIa treatment modality—meaning that there is conflicting evidence and/or divergence of opinion about its efficacy, but the weight of evidence is in favor of usefulness for this purpose [4], [97], [109]. In fact, the American Society of Regional Anesthetics (ASRA) has recommended ILE as a first-line treatment in local anesthetic systemic toxicities and not just as rescue therapy [97]. The American Heart Association (AHA), in its 2015 guidelines related to cardiovascular resuscitation and emergency cardiovascular care, adopted a more conservative approach. They endorsed ILE therapy only for bupivacaine overdose and for rescue therapy in other circumstances of cardiopulmonary arrest [77].

## 4 Safety of ILE

Reports of complications, especially in neonates, following the administration of large volumes of highly concentrated ILE include sepsis, lipid deposition in the pulmonary vasculature, increased pulmonary artery pressure, the development of chronic lung disease, and a reduction in arterial oxygen concentration during infusion [69], [91], [125], [137], [138], [139]. Importantly, such events have not been reported in the early literature that described the use of ILE as an antidote in drug-induced poisonings [149]. A recent review cited 18 case reports (in 12 articles of the 94 included in the analysis) of adverse effects of ILE when given in exceptionally high doses as an antidote in poisoning [17]. These effects included extreme transient lipemia, pancreatitis, ARDS, and two cases of post-ILE cardiac arrest [16], [17], [27], [79], [152]. The causal relation to ILE infusion is not clear. However, it is advisable to monitor patients several hours after lipid-based resuscitation [17], [27].

Studies on the untoward effects of ILE when used for parenteral nutrition in patients with acute respiratory distress syndrome (ARDS) raised concerns of possible exacerbation of their condition [20]. Some studies demonstrated transient reduction in blood oxygenation and worsening of hemodynamic parameters during infusion of lipid emulsions containing long-chain or long chain/medium chain fatty acids in these patients (with ARDS) [69], [78], [86], [139]. However, others showed either no-effect or some improvement [44], [85], [119]. Of particular interest, the study of Hwang et al. [69] showed that ILE infusion did not affect oxygenation and pulmonary hemodynamics in patients with normal lung function and respiratory conditions like pulmonary infection and COPD. Also, these results were found to vary with the fatty acid composition of the ILE and speed of infusion. These variable responses were attributed to variable production of vasodilatory or vasoconstrictory prostaglandins [86], [120], [129]. Moreover, other reports of successful use of parenteral ILE in cases of overdose of verapamil [80], [123] buproprion, and amitriptyline [79], [124], showed that ARDS, when developed, was managed promptly and successfully without any recognized effect on the patient outcome.

ILE is not recommended as a rescue treatment during hypoxia [59], [64], [88], [146]. These observations come in accordance with results showing ILE in ischemia may worsen myocardial oxidative damage [7].

Besides issues of direct safety, there is some evidence of interaction between ILE and lipophilic drugs used for resuscitation, potentially narrowing their efficacy [18], [98]. It has been shown that high doses of epinephrine may impair the efficacy of ILE [64], while low doses of epinephrine may improve it [65]. Another caveat is the use of ILE early after ingestion of toxicants [22]. It has been shown that blood lipids may enhance gastrointestinal absorption of toxicants, thereby increasing their likelihood of systemic toxicity [36], [60], [83], [107]. It should be mentioned that the reports of successful use of ILE for resuscitation in cases of drug ingestion were primarily in severely symptomatic patients, likely to have already experienced significant distribution of the toxicant. In summary, although the safety issues and caveats discussed above are significant, ILE infusion should be entertained as a life-saving regimen in circumstances of otherwise fatal drug/chemical overdose.

## 5 ILE and OP poisoning

The fast progression-to-death of the organophosphate toxidrome warrants the use of antidotes as early as possible [116]. The antidotes include atropine as a muscarinic receptor antagonist, oximes as AChE reactivators, and benzodiazepines to stop seizures [43], [116], [122]. However, even after using currently available treatments (e.g., atropine, oximes, and benzodiazepines), victims often develop long-term sequelae, mainly learning and memory deficits, intermediate syndrome and OP-induced delayed neuropathy (OPIDN) [2], [34]. The availability of OPs and their devastating effects are the reason for on-going efforts in developing effective medical countermeasures. Many of the OPs are lipophilic compounds. It therefore appeared reasonable to determine whether ILE would be useful in OP poisoning [40], [141], [154].

So far, there have been several animal studies on the use of ILE in experimental OP poisoning, though with inconsistent results. Recently, Mir and Rasool [90] reported on successful reversal of severe OP-induced cardiotoxicity in human OP poisoning by Intralipid, which seems to be the first report of ILE therapy in human OP poisoning.

### 5.1 Lipophilicity of CWA and organophosphate pesticides

As ILE therapy was mainly based on scavenging of lipophilic compounds, studies aimed at predicting its efficacy correlated the lipophilicity of different agents, measured by the octanol–water partition coefficients (log *K*_ow_), with pharmacologically relevant properties like scavenging from blood [17], [51], [89]. The octanol–water partition coefficients of the prominent chemical warfare agents sulfur mustard (HD), VX, sarin (GB), and soman (GD) are listed in Table 1, together with some OP pesticides and drugs whose poisoning was treated successfully with ILE infusion for reference. The OP pesticides parathion and diazinon, as representatives of a group of highly-lipophilic toxicants, are somewhat more lipophilic than the “gold standard” bupivacaine. Their active metabolites diazo–oxon and paraoxon are less lipophilic and, if we adopt log *K*_ow_ > 2 as a criterion for high lipophilicity [17], they can be viewed as marginally lipophilic. The chemical warfare agents sulfur mustard and VX are of low lipophilicity (*K*_ow_ = 2.4 and 2.1, respectively). The lipophilicity of the G-type agent soman is marginal, while sarin is non-lipophilic. The relative low lipophilicity of the prominent chemical warfare agents does not preclude considering a role for ILE infusion in the emergency medical management, as successful application of ILE infusion was observed with non-lipophilic compounds like atenolol [20], [21], for which mechanisms other than lipid scavenging may be attributable.

**Table 1:** Partition coefficients (log *K*_ow_) of chemical warfare agents and selected toxicants [11], [51], [82], [93]. CWA, chemical warfare agents; OP, organophosphates.

Compound	Type	Log *K*_ow_
Amitriptyline	Tricyclic antidepressant	5.0
Parathion	OP pesticide	3.83
Diazinon	OP pesticide	3.81
Bupivacaine	Local anesthetic	3.4
Sulfur mustard	CWA, blister	2.41
Malathion	OP pesticide	2.4
VX	CWA, nerve	2.09
Diazo–	OP pesticide, active metabolite of diazinon	2.07
Paraoxon	OP pesticide, active metabolite of parathion	1.98
Mepivacaine	Local anesthetic	1.9
Soman (GD)	CWA, nerve	1.78
Sarin (GB)	CWA, nerve	0.3
Atenolol	Beta blocker	0.2
Glyphosate	OP herbicide	−4.0

### 5.2 In vitro studies

In an in vitro investigation, Von Der Wellen et al. [141] studied the interactions of clinically approved Intralipid emulsions with OP nerve agents (GA, GB, GD, GF, and VX) and pesticides (paraoxon–ethyl and malaoxon), using an AChE inhibition assay and looking at the OP degradation kinetics. The aim was to validate the physical rationale for in-vivo studies of ILE in OP poisoning. They found that the incubation of OP compounds in intravenous lipid emulsions resulted in stabilization of the OPs, and only a negligible effect on degradation was recorded [141]. Though in this in vitro study an interaction of the OPs with the lipid phase was seen, it showed no evidence for a beneficial effect via modulation of OP degradation.

### 5.3 Animal studies

Bania et al. [5] tested whether pretreatment with Intralipid could protect mice from lethal exposure to paraoxon (administered 20 min following ILE). The results showed small, statistically insignificant increase in the LD50, time-to-onset of symptoms, and time-to-death in ILE-treated animals compared to saline controls, and were, hence, inconclusive. In the study of Moshiri et al. [93], rats were treated with ILE after oral exposure to diazinon and followed for 48 h. Again, the use of ILE failed to reduce mortality or increase survival time when compared with normal saline. Dunn et al. [36] also showed that ILE infusions starting five minutes after oral exposure to parathion had no positive clinical effect. However, when started 20 min after exposure, it prolonged the time-to-apnea, although no difference was shown in the numbers of animals with respiratory arrest. Because of the complex toxicokinetics of parathion, the reported effects of lipid infusion in the latter study are difficult to explain. However, it is possible that lipid scavenging may be involved [36], and this study emphasizes the importance of lipid infusion timing relative to the peak blood concentration of the toxicant [36]. The timing of ILE administration, which is likely to significantly affect its efficacy in oral intoxication, was apparently overlooked in acute studies of Moshiri et al. [93], but was fundamental in the experimental design of Dunn et al. [36], who selected the ILE starting times to represent the time points of initial and peak blood parathion concentrations after oral dosing. On an ironic note, the delay in onset of some critical toxic effects may allow the caregiver enough time to use currently available antidotes and supportive care.

In these above animal studies ILE was given alone, and not in conjunction with currently approved OP antidotes. Since OPs exert their effect relatively fast, it is advisable to perform studies to test the synergistic effect of ILE with the known antidotes, before ruling out its use. Such a study was performed by Kayipmaz et al. [73], in which rats were exposed to the lipophilic OP methyl parathion through intragastric gavage, and treated with the accepted combination of antidotes, including repeated doses of atropine for a total of 12 h, an oxime reactivator (pralidoxime). One of the groups was also treated with ILE (detailed protocol1 in Kayipmaz et al. [73]). Animals treated with ILE were found to have reduced brain, liver, and pancreatic injury when compared with animals treated with the accepted antidotes alone. More specifically, the authors described reduced expansion of glial cell feet following treatment with ILE, suggesting protection of the blood–brain barrier. Since the animals were exposed to a sub-lethal dose of methyl parathion, animals did not suffer from respiratory failure, convulsions, cardiac arrest or loss of consciousness. However, the authors indicated that the animals were extremely agitated upon receiving the ILE treatment.

### 5.4 Human case report—reversal of cardiovascular failure in suicidal parathion poisoning by i.v. administration of intralipid

Recently, Mir and Rasool [90] reported the case of a severely OP-poisoned patient that, on admission, presented with seizures, bradycardia, restlessness, miosis, hypersecretion, hypotension and respiratory insufficiency. She was decontaminated, intubated and ventilated, received fluid resuscitation, and was treated with repeated doses of atropine and pralidoxime according to the World Health Organization (WHO) protocol. The seizures were treated with phenytoin. Laboratory tests revealed severe acidosis, hypoxia, hypercapnia and a low level of plasma ChE. Chest X-ray was suggestive of ARDS. The patient had poor response to both antidotes and to supportive treatment, necessitating the initiation of vasopressor support. Despite the use of vasopressors, bradycardia with hypotension continued, followed by the onset of arrhythmias with fast ventricular rate, QT prolongation progressing to ventricular tachycardia (VT) and ventricular fibrillation within several hours of exposure. Standard cardiopulmonary resuscitation efforts including repeated cardioversion and the use of amiodarone and high-dose inotrope drugs failed to re-establish an effective cardiac rhythm. As repeated attempts of standard cardiopulmonary resuscitation failed, boluses of 20% intralipid were given i.v. up to a total of 300 mL, with the concomitant disappearance of extrasystoles, decreased QRS duration, and restoration of sinus rhythm. The need for vasopressor support and atropine started gradually to decrease, parallel with improvement in level of consciousness and resolution of respiratory distress. As expected, plasma ChE began to rise after the 3rd day and returned to normal values on the 7th day. On the 5th day of admission the patient was extubated, and by the 7th day atropine was discontinued. The authors attributed the successful reversal of the cardiotoxicity to sequestration of the highly-lipohilic parathion in a lipid scavenging mechanism, although direct cardiotonic and cardioprotective mechanisms, that were found to be as important in other instances of drug-induced cardiotoxicity [46], [47], [48], [76], [103], [131], cannot be ruled out, especially if the active agent may be the more potent and less lipophilic metabolite paraoxon.

### 5.5 Other routes for lipid emulsion treatment in OP poisoning

#### 5.5.1 Oral administration

Tuzcu et al. [132] treated malathion-exposed rats (p.o.) with an oral lipid emulsion (ILE given p.o.), and in this preliminary study showed that the oral emulsion prevented OP-induced pancreatic injury, specifically β-cell injury, if given up to six hours post-exposure. When giving the emulsion 12 h post exposure no effect was noted. Another group also looked at the effect of oral lipid emulsion in rats following oral exposure to malathion or chlorpyriphos, both lipophilic compounds, in different time points [12], [101]. Since it was shown in the past that OP exposure leads to oxidative stress [1], [71], [92], [106], [111], [140], this group decided to focus on total antioxidant capacity (TAC) and total oxidant status (TOS) of the rat brain, as well as on immunohistochemical staining of caspase-3 as a marker for apoptosis. In the malathion study, lipid emulsion was given either immediately or with a delay of six and 12 h post-exposure [12]. In the malathion-poisoned animals, TOS levels were significantly increased and TAC levels reduced compared to controls and immediate lipid-treated animals. However, there was no significant difference in TAC levels between the malathion-poisoned untreated group and delayed (six and 12 h post-exposure) lipid-treatment groups, but TOS levels were reduced in all lipid-treated groups. These results were consistent with levels of caspase-3. In the chlorpyriphos study, lipid emulsion was shown to have a protective effect on serum ChE, it increased TAC and decreased TOS, and when compared with the chlorpyriphos-only group, apoptosis was reduced in the lipid-treated groups [101].

Though these studies might show the early use of lipid emulsions in OP poisoning as promising, they have limitations: first, ILE was not tested together with other antidotes, and second, the authors were looking mainly at the antioxidant capacity and apoptosis, and not on other clinical aspects of poisoning. As exposure levels were below the lethal and even the acute clinical toxicity range, these results, as well as the oral administration of ILE, may be relevant for the low-level, chronic exposure scenarios or long-term effects in survivors of acute poisoning.

#### 5.5.2 Intraosseous administration

Severely OP-poisoned victims usually suffer from respiratory insufficiency combined with cardiovascular collapse [116]. Even when not in the setting of a mass casualty event, these patients pose a significant challenge to caregivers [121]. We, and others, have previously shown that the intraosseous route is an important method to administer relevant OP antidotes [13], [15], [41], [95], [135], [136]. Recently, Fettiplace et al. [45] have shown that intraosseous lipid emulsion can be safely used in emergency situations. This is an important issue when dealing with drug administration during resuscitation of a poisoned victim. Together with the data we have on the use of OP antidotes via the intraosseous route, it opens a potential treatment modality which may be beneficial in victims of acute, high-level OP poisoning.

The study of Bania et al. [5] on the effect of ILE in OP poisoning was based on intraperitoneal (i.p.) administration. Further studies are necessary in order to determine the differences in pharmacokinetics and bioavailability of the ILE delivered i.p. versus the more direct intravenous route.

### 5.6 The relevance of ILE in the management of OP poisoning

Even though the few reported studies on the role of ILE in OP poisoning did not show dramatic results concerning the efficacy of ILE in rescue of poisoned animals, the role of ILE in OP poisoning refractory to conventional treatment should not be minimized as evidenced by the report of Mir and Rasool [90].

Mechanistic considerations discussed below strengthen this point and justify further research efforts to establish its appropriate place in the treatment plan.

The mechanisms-of-action of ILE discussed in this manuscript address more than a single pathophysiological route of nerve agent or OP pesticide poisoning, as follows:

• “Lipid shuttle”: ILE was shown to extract lipophilic toxicants from critical organs like the brain and the heart [63]. Understanding of the pharmaco/toxicokinetics of the agent in different exposure modalities is essential in order to identify the optimal timing of ILE administration. The “lipid shuttle” mechanism may be relevant in the case of the CWAs VX and soman due to their lipophilicity and PK/PD properties. The distribution of VX is slow, so that lipid trapping (by ILE) may be efficient in hampering its accumulation in essential organs like the brain or heart. The unique property of soman is the rapid “aging” of the soman-bound ChE that stabilizes the soman-ChE bond making it refractory to reactivation by oximes [121], [122]. Therefore, sequestering of unbound soman is important in order to stop it from further binding to cholinesterase and subsequent ageing.
• Immunomodulatory and anti-inflammatory properties: inflammatory processes in the CNS are pivotal in the pathology of OP poisoning [9], and in the lung injury that is frequently-associated with such poisoning [67]. Recently, the ω-3 polyunsaturated fatty acid α-linolenic acid was shown to be neuroprotective in soman-poisoned rats [102]. The neuroprotective action of this fatty acid is pleiotropic in nature and the known anti-inflammatory properties of this and other ω-3 polyunsaturated fatty acids may play part in neuroprotection [110].
• Cardioprotective and hemodynamic properties: these may be beneficial in the control of OP-associated pulmonary and cardiac complications [3], [10]. This was suggested by recently-reported reversal of severe OP-induced cardiotoxicity in a human patient by ILE [90].
• Mitochondrial protection, antiapoptotic and antioxidant properties: poisoning with OP pesticides in humans and with CWAs in animals are associated with increased oxidative stress [1], [71], [92], [106], [111], [140] and with tissue damage, apoptosis and necrosis of cells in the CNS and in other organs [29], [74], [87]. Mitochondrial dysfunction in the CNS and other tissues, which was found to occur in OP pesticide poisoning [74] and in nerve-agent exposure [28], has a causal role in the elicitation of oxidative imbalance and apoptosis. In vitro studies of the effects of OP pesticides have shown that their apoptotic effects are mediated via the mitochondrial signaling pathway and driven by oxidative rather than cholinergic processes [74]. In the CNS, neuronal apoptosis is a late effect, post-cholinergic, resulting from seizures [29]. Studies in temporal lobe epilepsy (TLE), a condition that has many pathological features in common with acute nerve agent poisoning [29], [122], have shown that mitochondrial dysfunction may be a causal factor in the generation of seizures [50]. Thus, the beneficial effects of long-chain fatty acids in mitochondrial dysfunction [103], [112], [134] may have a role in alleviation of toxic effects in cases of OP poisoning, including seizure control. The recently published LIPAEMIC article, reported on seven out of 10 bupivacaine-poisoned individuals who developed seizures. These seizures were terminated by ILE treatment, and only two of the patients received an additional anticonvulsant (midazolam) [24]. Although the pathophysiology of seizures in bupivacaine and OP poisoning may not be the same, the role of ILE in sequestering the proconvulsive agents thus preventing exposure of target tissues warrants further study of their usefulness as anticonvulsants in OP poisoning as well.

## 6 Potential clinical use and future directions

Sequestration of nerve agents in the blood by scavenging molecules is an established strategy driving comprehensive research and development efforts [96]. Current bioscavengers under development are based on high-affinity binding proteins like AChE, BuChE, and praoxonase-1 (PON1) [96]. However, most of these candidate therapies have not been tested clinically and are not yet available for use. The ILEs, even though less potent as OP binders than specific OP targeted agents, are available in large quantities, have been used clinically for other purposes, and may be useful in entrapment of the highly lipophilic nerve agents soman and VX, as well as other lipophilic OP compounds, thus affecting their toxicity by interference with their distribution to the tissues. The lipophilic nature of sulfur mustard, together with the latent period from exposure to the development of tissue injury, makes ILE an important potential candidate as a medical countermeasure.

Exposure to OPs, whether intentional or accidental, leads to the death of several hundreds of thousand people every year [43], [55]. In a mass casualty event involving toxic agents, medical response assets should not only be effective, but they should also be readily available and simple to deploy. As is shown in this review, ILE may be an efficient broad-spectrum antidote, which exerts its antidotal properties in a rapid manner. At present, ILE therapy is intended exclusively for hospital use. However, as it does not impose a heavy logistic and operational burden, it is very easy to administer, is easily packaged and needs no refrigeration, it is very easily adaptable for prehospital emergency care.

In the hospital setting, no matter how many casualties are involved in an OP poisoning scenario, there might be an important role for ILE as an adjunct to currently accepted antidotal treatment. ILE may be useful in the management of sulfur mustard exposure, of which the toxic mechanisms are less well-defined and specific treatments are still a remote option. As it shares a number of physicochemical and toxicodynamic properties with nerve agents, some common treatment modalities for both types of chemical agents may be found [30], [31].

In any case, before we can decide on the role of ILE in the treatment of CWA poisoning, we should direct and conduct a methodical research program aimed at understanding better its mechanism-of-action in OP and sulfur mustard poisonings, study better its PK/PD, routes and timing of administration, and interactions with currently used antidotes in order to design combination therapies. Another important research direction is the effect of different types and compositions of ILE, especially those enriched in ω-3 polyunsaturated fatty acids, shown recently to be superior to standard ILE preparations in the protection of ARDS patients [119], [120] and in animal models [62], and, as cited above, may be useful in amelioration of nerve agent neuropathological effects2 [102], [110].

## 7 Summary and conclusions

Since many OP compounds and sulfur mustard are li pophilic, ILE might represent a new approach to the treatment of such poisonings. In several animal studies with OP pesticides, protection was not achieved, while other studies like that of Dunn et al. [36] showed some changes in the pathophysiology that merits additional studies. The successful reversal of cardiac arrest following ILE treatment in a human case of severe parathion poisoning, also suggests ILE may have a role in the management of severe OP poisoning. ILE was not studied so far in the treatment of sulfur mustard injury, and its potential role in the management of this injury should be explored. ILE may help in reducing the time to recovery of patients, by facilitating the extraction of the poison from the blood and tissues, by adding a large energy supply to the injured tissues, as well as other agent-dependent pharmacodynamic effects.

An important consideration in establishing a role for ILE in CWA poisoning is its availability in every hospital, the medical staff is acquainted with its use, and it is of relatively low cost. All of the above may warrant it as a simple, available, safe and cheap adjunct in the medical management of OP and CWA poisonings.

## Transparency document
